# Generation of Novel Bone Forming Cells (Monoosteophils) from the Cathelicidin-Derived Peptide LL-37 Treated Monocytes

**DOI:** 10.1371/journal.pone.0013985

**Published:** 2010-11-15

**Authors:** Zhifang Zhang, John E. Shively

**Affiliations:** Department of Immunology, Beckman Research Institute of the City of Hope, Duarte, California, United States of America; Universität Würzburg, Germany

## Abstract

**Background:**

Bone generation and maintenance involve osteoblasts, osteoclasts, and osteocytes which originate from unique precursors and rely on key growth factors for differentiation. However, an incomplete understanding of bone forming cells during wound healing has led to an unfilled clinical need such as nonunion of bone fractures. Since circulating monocytes are often recruited to sites of injury and may differentiate into various cell types including osteoclasts, we investigated the possibility that circulating monocytes in the context of tissue injury may also contribute to bone repair. In particular, we hypothesized that LL-37 (produced from hCAP-18, cathelicidin), which recruits circulating monocytes during injury, may play a role in bone repair.

**Methods and Findings:**

Treatment of monocytes from blood with LL-37 for 6 days resulted in their differentiation to large adherent cells. Growth of LL-37-differentiated monocytes on osteologic discs reveals bone-like nodule formation by scanning electron microscopy (SEM). In vivo transplantation studies in NOD/SCID mice show that LL-37-differentiated monocytes form bone-like structures similar to endochondral bone formation. Importantly, LL-37-differentiated monocytes are distinct from conventional monocyte-derived osteoclasts, macrophages, and dendritic cells and do not express markers of the mesenchymal stem cells (MSC) lineage, distinguishing them from the conventional precursors of osteoblasts. Furthermore, LL-37 differentiated monocytes express intracellular proteins of both the osteoblast and osteoclast lineage including osteocalcin (OC), osteonectin (ON), bone sialoprotein II (BSP II), osteopontin (OP), RANK, RANKL, MMP-9, tartrate resistant acid phosphatase (TRAP), and cathepsin K (CK).

**Conclusion:**

Blood derived monocytes treated with LL-37 can be differentiated into a novel bone forming cell that functions both in vitro and in vivo. We propose the name monoosteophil to indicate their monocyte derived lineage and their bone forming phenotype. These cells may have wide ranging implications in the clinic including repair of broken bones and treatment of osteoporosis.

## Introduction

Bone generation, maintenance and healing are complicated processes in which osteoblasts, osteoclasts, and osteocytes are known to play important roles. Osteoblasts, which are derived from mesenchymal stem cells (MSCs) [1], express marker genes for bone sialoprotein (BSP) and osteocalcin (OC). Osteoclasts, which are derived from monocytes by the action of macrophage colony-stimulating factor (M-CSF) and receptor activator of nuclear factor κB ligand (RANKL), effect bone resorption by removing its mineralized matrix and breaking up the organic bone [2]. In addition to these two types of cells, the bone contains osteocytes which are trapped in the bone matrix and cease to generate osteoid and mineralized matrix. The function of osteocytes is considered as inactive osteoblasts or bone lining cells [3] that undergo programmed cell death [3], [4]. However, the origin of bone forming cells during wound healing is less well studied. An unfilled clinical need of increasing bone mass such as nonunions of bone fracture indicated that unknown types of cells are involved in bone formation [5]. In the case of wound repair, monocytes play a critical role in the formation of new blood vessels [6], [7], [8], [9] and often differentiate into tissue specific lineages as required by the tissue of injury [10], [11]. Given the known plasticity of monocytes in wound repair, we speculated that monocytes may also contribute to bone repair not only as monocyte-derived bone absorbing osteoclasts, but also as yet to be defined bone forming cells. In order to investigate this possibility, we examined the response of monocytes to well known effectors of wound repair.

Among the many cytokines, chemokines, and other effectors associated with wound repair, hCAP-18 stands out as candidate with multiple effects on monocytes including the induction of migration [12], [13] and differentiation of monocytes to dendritic cells (DCs) [14]. hCAP-18 is the only human member of the cathelicidin family of antimicrobial peptides known to date [15]. LL-37, the proteolytically (proteinase 3) active product of hCAP-18, has been demonstrated to mediate a wide variety of immune and inflammatory functions, including wound repair. In this respect, growth factors involved in the process of wound healing were shown to induce LL-37 expression [16]. In vitro, LL-37 was shown to activate endothelial cells resulting in increased proliferation and formation of vessel-like structures, while in vivo cathelicidin (CRAMP)-deficient mice showed decreased vascularisation during wound repair [17]. LL-37 promotes re-epithelialization of healing skin [18] and anti-LL-37 antibodies inhibit re-epithelialization of skin wounds [19]. Furthermore, LL-37 induces migration of human peripheral blood monocytes, neutrophils, CD4^+^ T cells, and mast cells [12], [13], [20], [21] and influences the expression of over 40 genes in murine RAW 264.7 macrophage cells, in which some of these gene products are involved in bone formation, including the bone morphogenetic protein (BMP) 1, BMP-2, and BMP-8a [22]. Since LL-37 release increases during inflammation including trauma and bone fracture and induces several BMPs, we were interested in investigating the action of LL-37 on monocytes and their potential role in new bone formation.

We show that LL-37 treated monocytes form large adherent round cells after 6 days in culture, followed by the formation of large adherent round and irregular cells after 63 days in culture. Growth of LL-37-differentiated monocytes on osteologic discs for 7 weeks reveal bone-like nodule formation by scanning electron microscopy (SEM) and in vivo transplantation studies in NOD/SCID mice show that LL-37-differentiated monocytes form bone-like structures similar to endochondral bone formation. Moreover, LL-37-differentiated monocytes are distinct from monocyte-derived macrophages, dendritic cells (DCs), and osteoclasts according to their morphology, surface markers, and/or cytokine profiles. In addition, they do not express surface markers of the MSC lineage, clearly distinguishing them from the conventional precursors of osteoblasts. Interestingly, LL-37-differentiated monocytes express proteins of both the osteoblast and osteoclast lineage including osteocalcin (OC), osteonectin (ON), bone sialoprotein II (BSP II), osteopontin (OP), RANK, RANKL, MMP-9, tartrate resistant acid phosphatase (TRAP), and cathepsin K (CK). Since our data indicates that LL-37-differentiated monocytes are a new type of bone forming cell we propose the name monoosteophil. The ability to culture these cells in vitro may have wide ranging applications in the clinic including augmenting repair of broken bones and treatment of osteoporosis.

## Methods

### Reagents

Recombinant human GM-CSF, M-CSF, IL-4, IFNα, and RANKL were purchased from ProSpec Tany TechnoGene Ltd (Rehovot 76124, Israel); LPS from E. coli (055:B5), Phalloidin, and saponin from Sigma-Aldrich (St. Louis, MO, USA); anti-DC-SIGN PerCP/Cy5.5 (CD209, clone 9E9A8), anti-CD1a PE/Cy5(Clone HI149), anti-CD90 PerCP/Cy5.5 (Clone 5E10), anti-CD163-PerCP/Cy5.5 (Clone GHI/61), anti-CD34 PerCP/Cy5.5 (Clone 4H11), and anti-MMP-9 (Clone F37P4A3) were purchased from Biolegend Inc (San Diego, CA92121); anti-alkaline phosphatase (ALP, clone B4-78), anti-osteocalcin PE (OC, Clone 190125), anti-osteonectin (ON, Clone 122511), anti- RANKL (TRANCE, Clone 70525), anti-RANK (Clone 80707 were purchased from R& D System (Minneapolis, MN 55413, USA); anti-bone sialoprotein II (BSPII, Clone ID1.2), anti-osteopontin (OP, clone AKm2A1), rabbit anti-human tartrate resistant acid phosphatase (TRAP) and anti-human cathepsin K (CK) polyclonal antibodies were from Santa Cruz Biotechnology Inc (Santa Cruz, CA. 95060); anti-CD1b FITC (Clone M-T101), anti-CD16-PerCP-Cy5.5 (Clone 3G8), anti-CD14-FITC (clone M5E2), isotype controls, and BioCoat™ Osteologic™ Disc were from BD Biosciences (Chicago, IL, USA); anti-CD1c FITC (Clone AD5-8E7) was from Miltenyi Biotec Inc ( Auburn, CA95602); anti-CD45 PE (clone HI30) was from eBiosciences (Dallas, TX75312); Alexa Fluor 488 goat anti-mouse IgG (H+L), Alexa Fluor 488 goat anti-rabbit IgG (H+L), and Alexa Fluor 488 chicken anti-goat IgG (H+L) were from Invitrogen Corp (Chicago, IL60690); Hard Set mounting medium with DAPI was from Vector Laboratories (Burlingame, CA, USA); EasySep® Human Monocyte Enrichment Kit was from StemCell Technologies Inc (Vencouver, Canada); FBS and human serum were from Irvine Scientific (Santa Ana, CA, USA); LL-37 was synthesized, prepared and stored as previous reports[13], [23]. Briefly, LL-37 was synthesized by the standard FMOC chemistry, purified by reversed phase HPLC, and the mass verified by nanospray mass spectrometry. The concentration was determined by amino acid analysis.

### Monocyte preparation and differentiation

The use of anonymous discard blood samples without the requirement for informed consents was approved by the City of Hope IRB, IRB number 99132. Peripheral blood mononuclear cells (PBMCs) were isolated from citrated human blood (discard blood from anonymous donors) by centrifugation over Ficoll-Paque Plus (GE healthcare biosciences, Pittsburgh, PA, USA) density gradient. Monocytes were separated using EasySep® Human Monocyte Enrichment Kit (StemCell Technologies Inc, Vencouver, Canada) from PBMCs. The purified monocytes were stained with anti-CD14-FITC and checked by using flow cytometry FACSCanton II. Monocytes with >95% purity were suspended at 1×10^6^ cells/mL in RPMI 1640 medium supplement with 10% FBS (FBS contained <5 pg/100 mL LPS).

After isolation, monocytes were treated with serial dose of LL-37 (0.625 µM, 1.25 µM, 2.5 µM, 5 µM, or 10 µM) for differentiation for 3 days, 6 days, 14 days or more. After incubated for 6 days, cultures were fed every 2 days by removing half of the supernatant and adding fresh medium. For macrophage differentiation, monocytes were treated with medium only [24], with 100 ng/mL LPS [25], 10 ng/mL GM-CSF (macrophage 1, Mφ1), or 50 ng/mL M-CSF (macrophage 2, Mφ 2) for 6 days [26]. Monocyte-derived DCs were generated with 1,000 U/mL GM-CSF and 500 units/mL IL-4 [26] or GM-CSF and 1000 U/mL IFNα [27]. Osteoclast were differentiated from monocytes in the presence of RANKL and M-CSF (both at 25 ng/mL) [28].

### Cell lines

Clonogenic human MSCs were generously provided by Dr. Carlotta A. Glackin (Department of Neurosciences, City of Hope) and incubated in α-MEM supplemented with 20% fetal calf serum (FCS), 2 mM L-glutamine, and 100 µM L-ascorbate-2-phosphate as previously described [29].

### Microscopic observation of differentiated monocytes

Monocytes were plated in culture media and treated with LL-37 (dose shown in the figure), LPS (100 ng/mL), GM-CSF (10 ng/mL), M-CSF (50 ng/mL), GM-CSF (1,000 U/mL ) /IL-4 (500 units/mL), GM-CSF (1,000 U/mL ) /IFNα (1,000 U/mL ), or M-CSF/RANKL (both at 25 ng/mL) and photographed with the Leica DMI 3000B (Leica Microsystems Inc, Bonnockburn, IL60015) inverted microscope.

### Phagocytosis

6-day LL-37-, medium-, LPS-, GM-CSF-, and M-CSF-differentiated monocytes (1 × 10^6^/mL) in tissue culture medium supplemented with 10% FBS in a 24-well plate were mixed with fluorescent labeled latex beads at a multiplicity ratio of 1∶500, and incubated for 1 h at 37°C. After washed three times, cells were analyzed using FACSCanton II and Flowjo software.

### Flow cytometry

For cell surface staining, cells were washed with PBS, blocked with 10% human serum in PBS, stained with isotype controls and antibodies described in the figures, washed 3 times with 1% BSA PBS, and analyzed with a FACSCanton II. For intracellular staining, cells were washed with PBS, fixed with 1% paraformaldehyde in PBS, permeabilized in PBS buffer containing 0.1% saponin (Sigma-Aldrich) and 2% BSA, and stained with primary isotype controls and antibodies shown in the figures. After washing with PBS containing 1% BSA and 0.1% saponin, cells were stained with secondary Alexa 488 conjugated antibodies. Stained cells were assessed with a FACSCanton II cytometer, and analyzed using Flowjo software.

### Cytokine multiplex analysis

Fresh monocytes were plated at 1×10^6^ cells/mL in RPMI 1640 medium containing 10% FBS in 48-well plates and incubated with medium only, LL-37, LPS, GM-CSF, M-CSF, M-CSF/RANKL for 6 days, or/and 14 days and 21 days. Supernatant was collected and analyzed using the Human Cytokine 30-Plex antibody bead kit from Biosource International Inc, including IL-1β, IL-1ra, IL-2, IL-2R, IL-4, IL-5, IL-6, IL-7, IL-8, IL-10, IL-12, IL-13, IL-15, IL-17, TNFα, IFNα, IFNγ, G-CSF, GM-CSF, MIP-1α, MIP-1β, MIG, RANTES, eotaxin, MCP-1, IP-10, VEGF, HGF, FGF-basic, and HGF.

Cytokine concentrations were calculated using Bio-Plex Manager 3.0 software with an eight-parameter, curve-fitting algorithm applied for standard curve calculations [30].

### Bone resorption assay

The resorbing activity was evaluated by using BioCoat™ Osteologic™ Discs purchased from BD Biosciences as a mineral matter in 24-well plate. Monocytes were incubated with M-CSF/RANKL (both at 25 ng/mL) or 5 µM LL-37 on BioCoat™ Osteologic™ Discs for 21 days in 5% CO_2_ atmosphere, then cells were removed by exposure to NaOCl. The plates were washed by rinsing distilled water and pit formation was observed by light microscopy [31] and scanning electron microscopy (SEM). LL-37-differentiated monocytes and M-CSF/RANKL-differentiated osteoclasts were also incubated on BioCoat™ Osteologic™ Discs for 7 weeks and fixed with 2.5% glutaraldehyde in 0.1 M phosphate buffer for SEM.

### Scanning electron microscopy (SEM)

BioCoat™ Osteologic™ Discs fixed in 2.5% glutaraldehyde in 0.1 M phosphate buffer, pH 7.2 (buffer A) for 1 h from bone resorption assay were washed three times in buffer A, postfixed with 1% osmium tetroxide in buffer A, then dehydrated in graded ethanol. The 100% ethanol solution was then replaced by propylene oxide, and the cells were embedded in Eponate. Thin sections were stained with uranyl acetate and lead citrate and examined with a FEI TECNAI G2 electron microscope.

### In Vivo transplantation studies and histological examination

All animal research was conducted according to IACUC guidelines. This research was approved by the City of Hope IACUC animal welfare committee protocol number 09028. Approximately 5.0×10^6^ monocytes plus LL-37 (5 µM) were mixed with 40 mg of hydroxyapatite/tricalcium phosphate (HA/TCP) ceramic powder (Zimmer Inc, Warsaw, IN) and then transplanted subcutaneously into the dorsal surface of 10-week-old immunocompromised NOD/SCID mice for 7 weeks [29]. 5.0×10^6^ monocytes alone, LL-37 (5 µM) alone or blank control were mixed with 40 mg of hydroxyapatite/tricalcium phosphate (HA/TCP) ceramic powder as controls. Harvested implants were fixed in 10% formalin neutral buffered solution, decalcified in 10% EDTA, and embedded in paraffin. Four-micrometer-thick sections were stained with hematoxylin and eosin, and trichrome. For immunohistochemistry, sections were stained usingprimary mouse anti-human BSP II monoclonal (Santa Cruz Biotechnology Inc) and secondary biotinylated rabbit anti-mouse IgG.

### Statistical analysis

Assay results are expressed as means±SE and unpaired Student's t-tests were used for comparisons. All p-values are two-sided. Data were analyzed with SPSS software (release 10.0, SPSS, Chicago, IL, USA) and GraphPad Prism software (version 5.0, GraphPad Software, San Diego, CA, USA).

## Results

### LL-37 induces monocyte differentiation and survival

**Figure 1:**
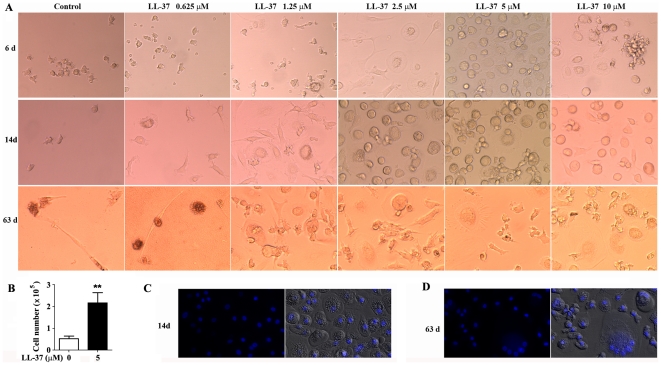
LL-37 induces monocyte differentiation. Negatively isolated monocytes at the concentration of 1×10^6^/mL were incubated in RPMI 1460 medium with 10% FBS in the presence or absence of LL-37. **A**. Cell morphology was shown by phase contrast microscopy (magnification 200×). **B**. Monocytes were incubated in the presence or absence of 5 µM LL-37 for 6 days. Cells were harvested and counted by hemocytometer. Data (mean ± SE) was from four independent experiments performed. ** p<0.01 vs control. **C–D**. Monocytes treated with 5 µM LL-37 for 14 days (**C**) or 63 days (**D**) were fixed, permeablized, stained with DAPI, and observed by fluorescent microscopy. Multinucleated giant cell formation was found in 63-day LL-37-treated monocytes. Images represent one of four independent experiments.

After treatment with LL-37 for 6 days, monocytes differentiated into large round adherent cells in comparison with untreated cells that remained as either small round suspended cells or underwent cell death (**Figure 1A**). The numbers of 5 µM LL-37-treated cells are significantly higher than untreated controls in agreement with loss of cells in untreated controls by cell death (**Figure 1B**). The maximum effect occurred at the dose of 5 µM LL-37 after which higher doses (≥10 µM) led to decreased cell numbers due to the well known toxicity of LL-37 >10 µM [23], [32]. After 14 days, the cell number of untreated controls decreased further, but the LL-37-differentiated monocytes survived with an apparent further increase in the diameter of the round adherent cells. DAPI staining showed that LL-37-differentiated round adherent cells have a single nucleus in spite of their larger cell size (**Figure 1C**). However after continuous culture for 63 days some of the large round LL-37-differentiated monocytes became giant cells with a multinucleated characteristic similar to osteoclasts (**Figure 1D**). In addition to the large round cells, we noted the appearance of irregular shaped cells at 63 days for cells treated with 1.25–10.0 µM LL-37 (**Figure 1D**). Together these analyses indicate that LL-37 can progressively differentiate monocytes into large round adherent cells at early times with giant cells and irregular shaped cells appearing at later times.

### LL-37-differentiated monocyte has the ability of bone formation in vitro

**Figure 2:**
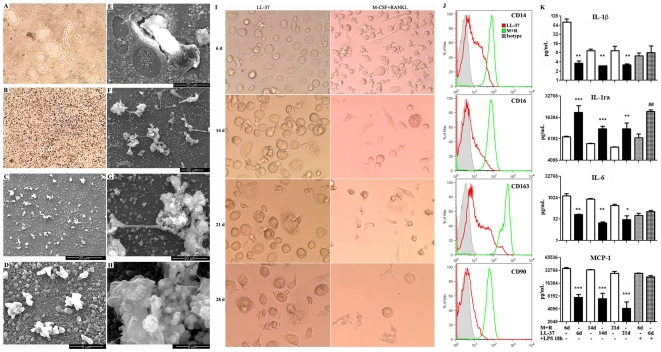
LL-37-differentiated monocytes are a novel type of bone forming cells (monoosteophils). Monocytes were incubated in the presence of M-CSF/RANKL (both at 25 ng/mL, **A**, **E**) or 5 µM LL-37 (**B,C,D,F,G,H**) on BioCoat™ Osteologic™ Discs in 5% CO_2_ atmosphere. After incubation for 3 weeks, cells were removed with bleach and observed by phase contrast microscopy (magnification ×200, **A**, **B**) or scanning electron microscopy (SEM) (**C**, **D**). Pit formation was shown on the disc incubated with M-CSF/RANKL-differentiated monocytes (**A**). Refractile specks were shown on the disc incubated with LL-37-differentiated monocytes (**B**). SEM showed the refractile specks are built-up granules with a shallow absorbed zone (**C**, **D**). After incubation for 7 weeks, pit formation and osteoclast are shown on the disc incubated with M-CSF/RANKL-differentiated monocytes by using SEM (**E**), and built-up structures and cells are shown on the disc of LL-37-differentiated monocytes (**F–H**). Monocytes at concentration of 1×10^6^/mL were incubated with 5 µM LL-37 or M-CSF/RANKL (both at 25 ng/mL). **I**. Morphology was evaluated by phase contrast microscopy (magnification 200×). **J**. After incubation for 6 days, LL-37- and M-CSF/RANKL-differentiated monocytes were harvested, stained with antibodies, and analyzed by flow cytometry. **K**. Cytokine levels in the supernatant of LL-37- and M-CSF/RANKL-differentiated monocytes were analyzed. In the LPS treated experiment, 6-day LL-37- and M-CSF/RANKL-differentiated monocytes were collected, resuspended at the concentration of 1×10^6^/mL, and incubated with 100 ng/mL LPS for 18 h and cytokine levels were detected in the supernatant (mean ± SE, n = 3). Data represent three independent experiments performed. M+R: M-CSF+RANKL. ** p<0.01, *** p<0.001 in comparison with M-CSF/RANKL-differentiated monocytes; ## p<0.01 in comparison with M-CSF/RANKL-differentiated monocytes treated with LPS for 18 h.

Culture of unfractionated peripheral-blood monocytes with M-CSF and RANKL is sufficient to induce their differentiation in vitro to osteoclasts [33]. The multinucleation characteristic of the osteoclast is the most striking morphological feature distinguishing the osteoclast from its monocyte precursor. Since LL-37-differentiated monocytes at late times have the morphology of giant cells of osteoclasts, it was likely that they would share their function, namely the dissolution/absorption of bone. BioCoat™ Osteologic™ Discs were used to evaluate the bone absorption ability of LL-37-differentiated monocytes compared to in vitro derived osteoclasts. After incubation on BioCoat™ Osteologic™ Discs for 3 weeks following removal of cells by bleach, M-CSF/RANKL-differentiated osteoclasts formed absorption pits on the BioCoat™ Osteologic™ Discs (**Figure 2A**) comparable to published results [31]. Unexpectedly, LL-37-differentiated monocytes formed refractile specks on the discs instead of classical absorption pits when viewed by phase contrast microscope (**Figure 2B**). Scanning electron microscopy (SEM) analysis revealed that the refractile specks are composed of raised granules on the surface of the BioCoat™ Osteologic™ discs (**Figure 2C**). Higher magnification analysis demonstrated single and compound granules on the discs with a shallow absorbed zone among compound granules (**Figure 2D**). These results prompted us to extend the incubation time from 3 weeks to 7 weeks and to include cells on the discs for SEM analysis (i.e, no removal of cells by bleach). As a control, M-CSF/RANKL-differentiated monocytes on the osteologic discs are shown with one osteoclast and two absorption zones (**Figure 2E**). In contrast, LL-37-differentiated monocytes exhibit raised granules on the discs with inclusion of granular material within the cells (**Figure 2F–H**). The identification of the granules as mineralized material was verified by positive von Kossa staining (**Figure S1, Supplementary Methods S1**). These results demonstrate that LL-37-differentiated monocytes are bone-forming cells, distinct from in vitro derived bone absorbing osteoclasts.

LL-37-differentiated monocytes were directly compared to monocyte-derived osteoclasts for morphology, survival, surface markers, and cytokine release. Our results showed that although LL-37-differentiated cells are morphological similar, they survive better than M-CSF/RANKL-derived osteoclasts after 28 days in terms of cell numbers (**Figure 2I**). While both treatments showed two adherent cell types, namely round large cells and irregular shaped cells at 28 days, there was a higher percentage of irregular shaped cells in osteoclast differentiation medium compared to LL-37 differentiation medium at 6 days. Surface marker staining showed that 6-day M-CSF/RANKL-treated monocytes expressed higher levels of CD14, CD163, CD16 and CD90 compared to 6-day LL-37-differentiated monocytes (**Figure 2J**). In addition, cytokine release analysis showed that LL-37-differentiated monocytes released lower levels of IL-1β, IL-6, MCP-1, but higher levels of IL-1ra than osteoclasts after differentiation for all time points analysed (**Figure 2K**). Furthermore, 6-day LL-37-differentiated monocytes showed increased production of IL-1ra, IL-10, IL-12, IP-10, and GM-CSF than 6-day M-CSF/RANKL-differentiated monocytes in response to LPS (**Figure** **2K** and **Figure S2**). The cytokine release profile in response to LPS suggests that LL-37-differentiated monocytes have an anti-inflammatory function. Thus, these data demonstrate that although LL37-differentiated cells share the morphology of giant cells similar to monocyte-derived osteoclasts, they have distinctive cell surface markers and cytokine release profiles.

Since LL-37-differentiated monocytes are not true osteoclasts, then perhaps they share properties with mesenchymal stem cells (MSCs) the precursors of osteoblasts? In the process of bone generation and repair, MSCs differentiate into osteoblasts and osteocytes. To answer this question, cell surface markers of both LL-37-differentiated monocytes and MSCs were compared. As expected, both MSCs and LL-37-differentiated cells don't express the bone marrow stem cell marker CD34. However, while MSCs express CD90 (Thy1), a marker for a variety of stem cells, they don't express CD45, a common leukocyte marker. In contrast, LL-37-differentiated monocytes express CD45 and do not express CD90 (**Figure S3**). Moreover, LL-37-differentiated monocytes exhibit both round enlarged and irregular cell shapes while MSC exhibit spindle shape morphology only (data not shown). Furthermore, when cultured under osteogenic inductive conditions on BioCoat™ Osteologic™ Discs, MSC exhibited a different pattern of bone formation compared with LL-37-differentiated monocytes as indicated by using SEM (**Figure S4**). Thus, we conclude that LL-37-differentiated monocytes, distinct from both osteoclasts and precursor of osteoblasts, are a new type of bone forming cells. In additional to the three types of bone cells, osteoclast from M-CSF/RANKL-derived monocyte, osteoblast from MSC, and osteocyte from MSC-derived osteoblast, we can now add LL-37-differentiated monocytes as a fourth type. We propose the name “monoosteophil” to distinguish them from the three known types of bone cells and to emphasize their bone loving behavior generated from monocytes.

### Bone formation of monoosteophils in vivo

**Figure 3:**
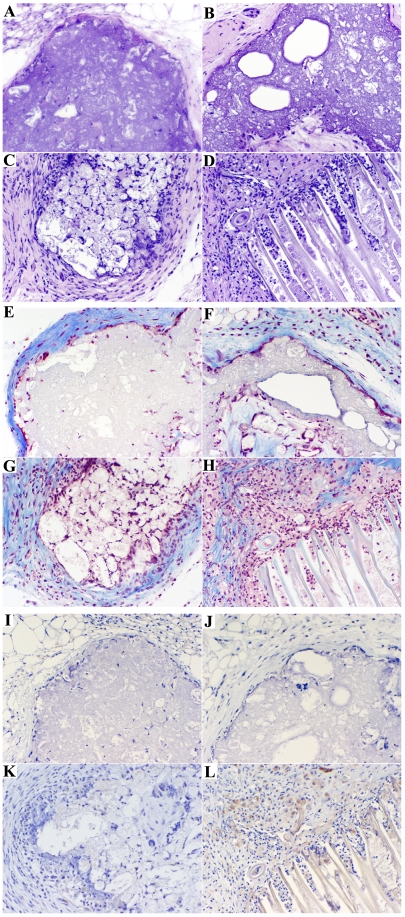
Bone formation of monoosteophils in vivo. NOD/SCID mice were implanted subcutaneously with hydroxyapatite/tricalcium phosphate only (**A,E, I**), or with 5 µM LL-37 (**B, F, J**), or with 5×10^6^ monocytes (**C,G,K**), or 5 µM LL-37 plus 5×10^6^ monocytes (**D,H,L**). Implants were harvested 7 weeks later and sections stained with haematoxylin and eosin (**A–D**), masson trichrome (**E–H**), and anti-human BSP II antibody (**I–L**) (100×).

The capacity of the monoosteophils to form ectopic bone when transplanted with hydroxyapatite/tricalcium phosphate (HA/TCP) ceramic particles subcutaneously in NOD/SCID mice was examined. Histologic analysis of hematoxylin and eosin (H&E) sections representing 7 week old transplants showed that monoosteophil implants did not stain red for bone matrix-like structures as observed for MSC implants [29]. An epiphyseal-like structure was observed only in the monoosteophil group but not in three control groups (monocytes alone, LL-37 alone, and blank control) (**Figure 3A–D**). Epiphyseal-like structures are usually seen in the proliferating layer of growth plates in the histologic stages of long bone and epiphyseal development [34], [35]. Masson trichrome staining showed that the epiphyseal-like structure contains collagen (blue color) (**Figure 3E–H**). When the origin of the cellular material in the recovered implants was assessed using anti-human BSP II antibody, the interstitial tissue and cells within the epiphyseal-like structure were found to stain positive for anti-human BSP II, confirming their human origin (**Figure 3I–L**). These results suggest that monoosteophils have the ability to form bone-like structures similar to the proliferating layer of growth plates.

### The monoosteophil is distinct from in vitro differentiated macrophages and dendritic cells

**Figure 4:**
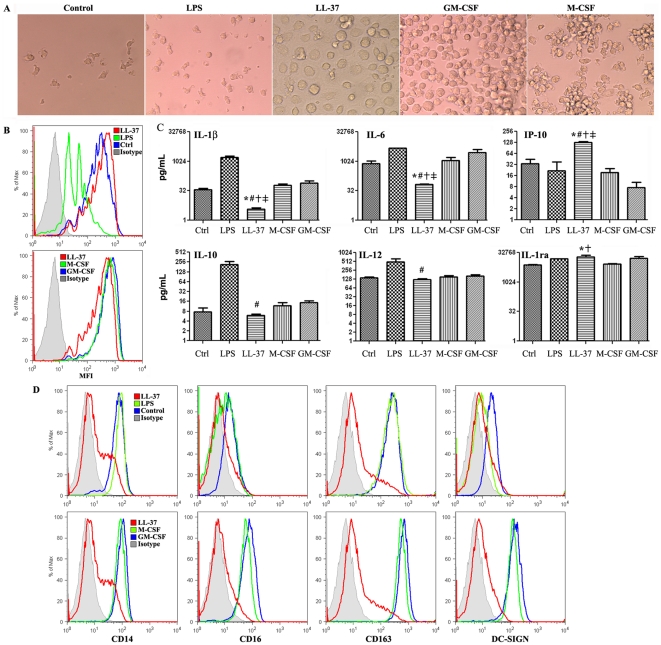
Comparison of monoosteophils with monocyte-derived macrophages. Negatively isolated monocytes at the concentration of 1×10^6^/mL were incubated in the presence or absence of 100 ng/mL LPS, 5 µM LL-37, 10 ng/mL GM-CSF, or 50 ng/mL M-CSF for 6 days. **A**. Cell morphology was shown by phase contrast microscopy (magnification 200×). **B**. 6-day medium-, LPS-, LL-37-, GM-CSF-, or M-CSF-differentiated monocytes at the concentration of 1× 10^6^/mL were mixed with fluorescent labeled latex beads at a multiplicity ratio of 1∶500, incubated at 37°C for 1 h, and analyzed by flow cytometry to show phagocytosis (MFI, mean fluorescence intensity). **C**. After monocytes were incubated with medium, LPS, LL-37, GM-CSF, and M-CSF for 6 days, cytokine/chemokine levels in cultured supernatant were analyzed (pg/mL, mean ± SE). **D**. 6-day medium-, LPS-, LL-37-, GM-CSF-, or M-CSF-differentiated monocytes were harvested, stained with antibodies, and analyzed by flow cytometry. Data shown were from three or four independent experiments.

Circulating monocytes have the capacity to differentiate into a variety of phagocytes, including macrophages, dendritic cells (DCs), osteoclasts, microglia in the central nervous system, and Kupffer cells in the liver [36], [37], [38], [39]. Thus, it was important to compare monoosteophils to monocyte-derived macrophage and DC lineages. At least 4 methods have been used to generate macrophages from monocytes, including incubation with medium only [24], LPS [25], GM-CSF to produce Mφ1, or M-CSF to produce Mφ2 [26]. As shown in **Figure 4A**, the morphology of LL-37-differentiated cells at 6 days is distinct from monocytes treated with medium, LPS, GM-CSF or M-CSF. Interestingly, a phagocytosis assay showed that the LL-37-differentiated cells retain their phagocytic function similar to the other generated macrophages except LPS-generated macrophage (**Figure 4B**), although this assay may not faithfully predict phagocytosis of opsonized bacteria. Cytokine profiles from the supernatants of 6-day LL-37 treated monocytes showed that LL-37-differentiated monocytes produce significantly less IL-1β and IL-6 and increased IP-10 in comparison with macrophages generated by the other methods (**Figure 4C** and **Supplementary Table S1**). Importantly, LL-37-differentiated monocytes release the highest levels of IL-1ra, the natural antagonist of IL-1β, and the lowest level of IL-1β compared to the other macrophage lineages. This suggests that LL-37-defferentiated monocytes do not function as inflammatory cells, at least according to inflammatory cytokine release, a result similar to our previous report that LL-37 induces IL-1ra release from neutrophils [23]. Moreover, cell surface marker staining showed that LL-37-differentiated monocytes expressed lower levels of CD163, DC-SIGN, and CD14 (**Figure 4D**) in comparison with the other macrophages lineages. Thus, although LL-37-differentiated monocytes retain their phagocytic ability, they are distinct from other in vitro generated macrophages in terms of cell morphology, cytokine release, and cell surface markers.

**Figure 5:**
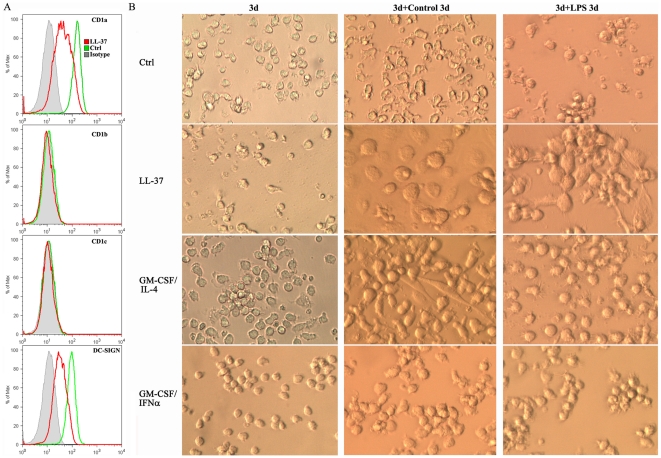
Monoosteophils are distinct from dendritic cells. Negatively isolated monocytes at the concentration of 1× 10^6^/mL were incubated in the presence or absence of 5 µM LL-37, 1,000 U/mL GM-CSF/500 U/mL IL-4, GM-CSF/IFNα (both at 1,000 U/mL). **A**. After incubation for 6 day, LL-37-differentiated monocytes were harvested, stained with antibodies, and analyzed by flow cytometry. **B**. Morphology of medium-, LL-37-, GM-CSF/IL-4-, and GM-CSF/IFNα-differentiated monocytes in the presence or absence of LPS were shown by phase contrast microscopy (magnification 200×). Images shown were from one of four independent experiments.

Besides the differentiation of monocytes along the macrophage pathway, monocytes can also differentiate into DCs when cultured in the presence of GM-CSF and IL-4 [40], [41] or GM-CSF and IFNα [27]. DCs are key players of adaptive immunity, unique in their ability to activate naïve T lymphocytes by operating as professional antigen-presenting cells (APC) to trigger a primary immune response. DCs are classified as immature prior to stimulation with agents such as LPS and as mature DCs after treatment with LPS, at which point they exhibit dendritic morphology [42], [43]. To investigate their interrelationships, LL-37-differentiated monocytes were compared to in vitro prepared DCs. As shown in **Figure 5A**, LL-37-differentiated monocytes express lower levels of CD1a and DC-SIGN than untreated controls and do not express CD1b and CD1c, typical markers of DCs [40]. The morphology of LL-37-differentiated monocytes is distinct from GM-CSF/IL-4- and GM-CSF/IFNα-derived immature DCs in culture for 3 or 6 days. After incubation with LPS for 3 days, GM-CSF/IL-4- and GM-CSF/IFNα-derived immature DCs showed mature dendritic morphology, a morphology not observed for LL-37-differentiated cells or untreated cells (**Figure 5B**). These results demonstrate that monoosteophils do not share the characteristics of monocyte-derived DCs regarding cell surface markers and cell morphology.

### Other characteristics of monoosteophils

**Figure 6:**
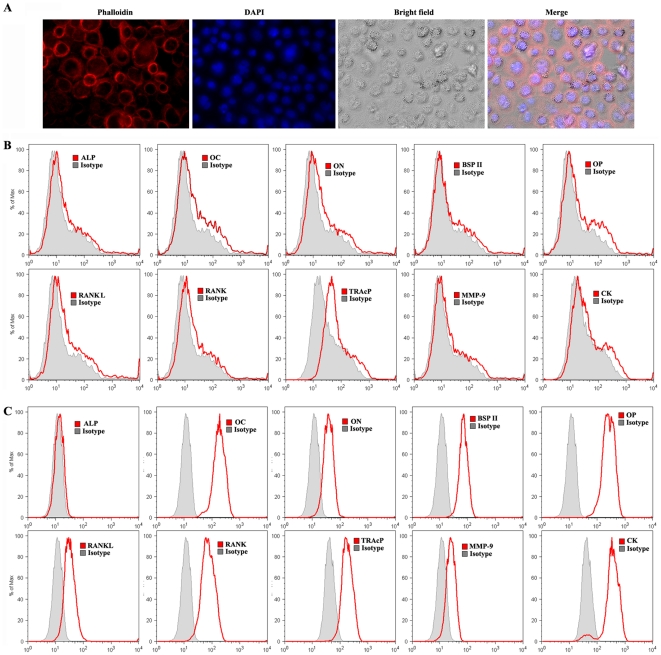
Characteristics of monoosteophils. Monocytes were incubated in the presence of 5 µM LL-37 for 6 days. **A**. cells were fixed, permeabilized, stained with phalloidin-Alexa 594 and DAPI, and observed by using fluorenscence microscope. **B**. cells were harvested, stained with antibodies, and analyzed by using flow cytometry to show surface staining of proteins. **C**. Cells were harvested, fixed and permeabilized, stained with antibodies, and analyzed by using flow cytometry to show intracellular staining of proteins. Data shown were from one of four independent experiments.

Given that monoosteophils are a novel type of bone forming cells, we performed additional studies to fully characterize their properties. Since monoosteophils have the ability of using existing bone material to build new bone, we expected that they would express markers of both osteoclast and osteoblast lineages. Indeed, phalloidin staining showed that after differentiation for 6 days most monoosteophils are round adherent cells with actin rings (**Figure 6A**), similar to osteoclasts but mononuclear. In addition, 6 day monoosteophils express a low level of TRAP, a marker normally expressed by bone-resorbing osteoclasts and activated macrophages, on their cell surface [44]. However, 6 day monoosteophils don't express other surface markers of osteoclasts including RANK, MMP-9, and cathepsin K (**Figure 6B**). In comparison to osteoblast markers, they do not express surface alkaline phosphatase (ALP), OC, osteonectin (ON), BSP II, or osteopontin (OP) (**Figure 6B**). Interestingly, intracellular staining results showed that 6-day monoosteophils do express most of the expected osteoblast and osteoclast proteins except ALP (**Figure 6C**), which is one of the most prominent markers of MSC-derived osteoblasts. Intracellular staining also reveals the presence of RANKL in LL-37 treated monocytes, suggesting that RANKL may be locally released by these cells. These data indicated that monoosteophils share the actin ring of the osteoclast, and express some marker proteins of the osteoclast and osteoblast but these marker proteins are localized to the cytoplasm and not to the cell surface.

## Discussion

Bone is a dynamic tissue that undergoes constant remodeling in which old bone is degraded by osteoclasts, the bone-resorbing cell, and subsequently replaced by new bone formed by osteoblasts, the bone-forming cell [33], [45], [46]. It is known that osteoblasts are derived from mesenchymal stem cells (MSCs) through a multi-step differentiation pathway [46]. Here, our data suggested that LL-37-differentiated monocytes (monoosteophils), which have different cell surface markers compared to MSCs, are a new type of bone forming cell. Moreover, monoosteophils express some characteristic proteins of both osteoblasts and osteoclasts.

Interestingly, both monoosteophils and osteoclasts are differentiated from monocytes. When monocytes are treated with M-CSF/RANKL, they differentiate into osteoclasts that exhibit a bone resorbing phenotype, but when monocytes are treated with LL-37, they produce monoosteophils with a bone formation phenotype. Thus, the environmental niche of monocytes may determine their ultimate phenotype. In the bone marrow where monocytes are exposed to M-CSF and RANKL, they become osteoclasts, but in an external wound-healing environment exposed to LL-37, they become monoosteophils. Circulating monocytes play an important role in development and homeostasis, in part via the removal of apoptotic cells and scavenging of toxic compounds [47]. Circulating monocytes also are accessory cells, linking inflammation and the innate defense against microorganisms to adaptive immune responses. In fact, circulating monocytes are a considerable systemic reservoir of myeloid precursors for the renewal of some tissue macrophages, antigen-presenting DCs [48], [49], [50]. Recently, it was reported that circulating monocytes have the potential to differentiate to a variety of cell types such as multipotential cells and endothelial cells other than phagocytes [10], [11]. For example, monocyte-derived multipotential cells (MOMC), having a unique phenotype positive for CD14, CD45, CD34, and type I collagen with a fibroblast-like morphology, can be obtained in cultures of peripheral blood mononuclear cells (PBMC) for 7 to 10 days on fibronectin-coated plastic plates supplemented with 10% fetal bovine serum as the only source of growth factors [10]. However, it should be noted that MOMC were isolated from PBMCs rather than from purified monocytes. Moreover, MOMC are spindle shape cells and monoosteophils contain both large round and irregular shaped cells. Furthermore, MOMC are CD14, CD45, CD34 positive, while monoosteophils are CD45 positive but CD14 and CD34 negative. Thus, monoosteophils are distinct from MOMC regarding generation, morphology, and surface markers.

LL-37 is a potent modifier of DC differentiation and maturation [14], [51], [52]. For example, monocytes that were co-cultured for 7 days with LL-37 and IL-4/GM-CSF were differentiated into DCs [14]; treatment of GM-CSF/IL-4-derived immature DCs with LL-37 and LPS suppressed the maturation and activation of DCs by TLR ligands [51]; GM-CSF/IL-4-derived immature DCs treated with LL-37 undergo internalization and subsequent localization of LL-37 in the cytoplasmic and nuclear compartments, causing an increased expression of HLA-DR and CD86 [52]. In our study, monoosteophils did not share dendritic cell markers and morphology with either GM-CSF/IL-4- or GM-CSF/IFNα-derived DCs. In another study, monocytes which were treated with either GM-CSF or M-CSF plus LL-37 differentiated to macrophages with an inflammatory phenotype [53]. Importantly, the main difference between our study and the other studies is that we used LL-37 as a sole factor for monocyte differentiation instead of combining LL-37 with other treatments which may have directed differentiation along another lineages.

Our results showed that LL-37 at the concentration of ≥1.25 µM ≤10 µM can induce monocyte differentiation. Importantly, these levels of LL-37 occur naturally during inflammation [54], [55], [56], [57], [58], [59]. So far, there is no report regarding the LL-37 concentration in the bone marrow or in bone undergoing repair. Since LL-37 is released during inflammation, our data suggest that the action of LL-37 on circulating monocytes may play an important role in bone fracture and repair, given that monoosteophils have the ability to use existing bone material to build new bone. We believe that subsequent studies will demonstrate a physiological and pathophysiological function for these cells. However, even in the absence of such studies, the in vitro production of these cells may have important implications for clinical situations demanding the rapid production of new bone in the case of unrepaired breaks or osteoporotic lesions.

In summary, our results indicate that LL-37-differentiated monocytes are a new type of bone forming cell, for which we propose the name monoosteophil. Because the processes of bone generation, remodeling, and repair are often compromised, the healing of fractures and treatment of diseases such as osteoporosis may benefit from the in vitro production of these novel bone forming cells from monocytes.

## Supporting Information

Figure S1 von Kossa staining of osteologic disc co-cultured with LL-37-differentiated monocytes. Monocytes were incubated in the absence or presence 5 µM LL-37 on BioCoat™ Osteologic™ Discs in 5% CO2 atmosphere. After incubation for 5 weeks, BioCoat™ Osteologic™ Discs were analyzed using von Kossa staining to demonstrate mineralization (dark color indicates mineral nodules). Original magnification: 200×.
(4.81 MB TIF)

Figure S2 Comparison of cytokine release between LL-37-differentiated monocytes and M-CSF/RANKL-differentiated osteoclasts. Monocytes were incubated in the presence of 5 µM LL-37 or M-CSF/RANKL (both at 25 ng/mL). Cytokine levels in the supernatant of LL-37- and M-CSF/RANKL-differentiated monocytes were evaluated using the Human Cytokine 30-Plex antibody bead kit. In the experiment with LPS treatment, 6-day LL-37- or M-CSF/RANKL-differentiated monocytes were collected, resuspended at the concentration of 1×10^6^/mL, and incubated with 100 ng/mL LPS for 18 h. Cytokine levels were detected in the supernatant. Data (mean ± SE) were from three independent experiments performed. ## p<0.01 in comparison with M-CSF/RANKL (M+R)-differentiated monocytes treated with LPS for 18 h.
(8.28 MB TIF)

Figure S3 LL-37-differentiated monocytes show surface marker differences from mesenchymal stem cells (MSCs). MSCs and 6-day 5 µM LL-37-differentiated monocytes (monoosteophils) were harvested, stained with antibodies, and analyzed by using flow cytometry and Flowjo software. Data represent one of three independent experiments performed.
(3.43 MB TIF)

Figure S4 Differentiation of MSCs on osteologic disc. MSCs were incubated in the α-MEM supplemented with 10% FCS, 100 µM L-ascorbate-2-phosphate, 10^−7^ M dexamethasone on BioCoat™ Osteologic™ Discs in 5% CO2 atmosphere. After incubation for 4 weeks, built-up structures and cells were shown using SEM (A). The region shown in the rectangle was magnified to show further details (B).
(1.89 MB TIF)

Methods S1 Supplementary methods.
(0.02 MB DOC)

Table S1 Comparison of cytokine release of LL-37-differentiated cells for 6 days with medium, LPS, GM-CSF, and M-CSF differentiated macrophages (Mean±SEM).
(0.05 MB DOC)
