# Supplementary figures and images for: Generation of Novel Bone Forming Cells (Monoosteophils) from the Cathelicidin-Derived Peptide LL-37 Treated Monocytes
Source: PLoS One. 2010 Nov 15;5(11):e13985. doi: 10.1371/journal.pone.0013985 (PMC2981577; doi:10.1371/journal.pone.0013985)

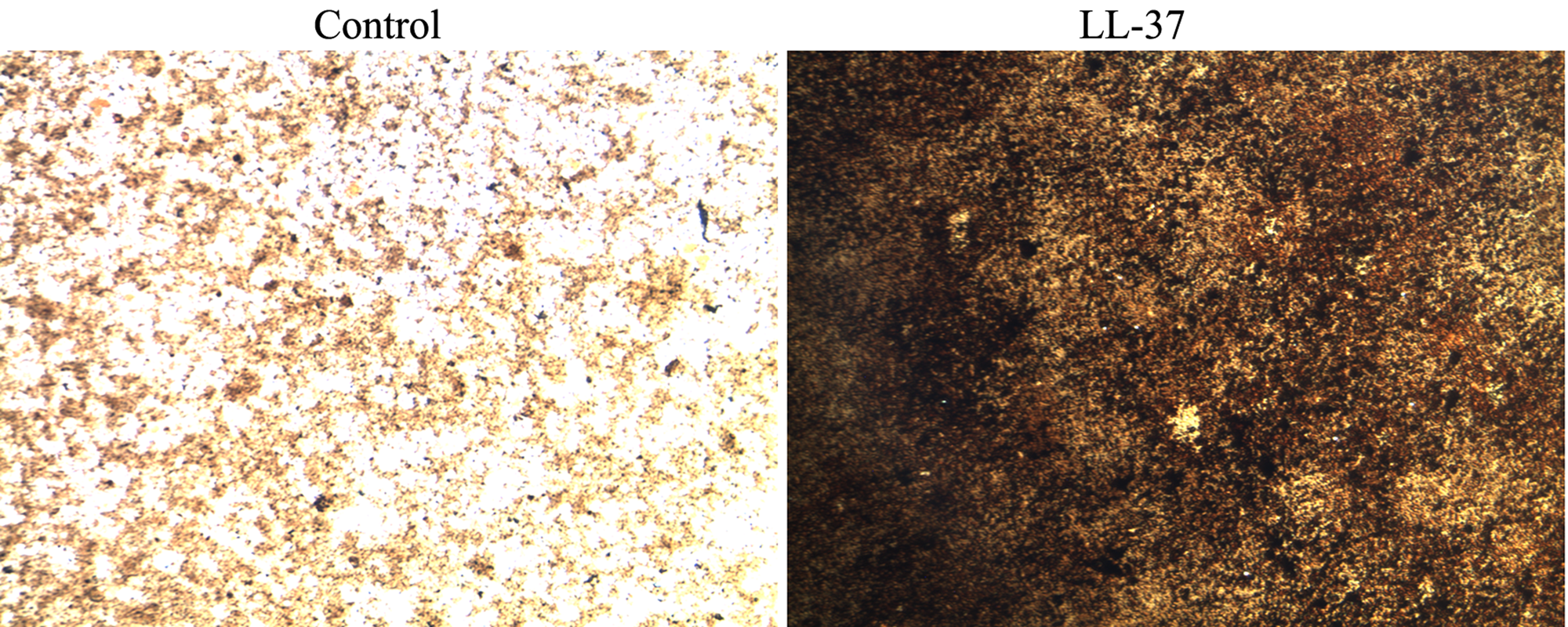

Supplement: Figure S1 — von Kossa staining of osteologic disc co-cultured with LL-37-differentiated monocytes. Monocytes were incubated in the absence or presence 5 µM LL-37 on BioCoat™ Osteologic™ Discs in 5% CO2 atmosphere. After incubation for 5 weeks, BioCoat™ Osteologic™ Discs were analyzed using von Kossa staining to demonstrate mineralization (dark color indicates mineral nodules). Original magnification: 200×. (4.81 MB TIF) [file pone.0013985.s001.tif]

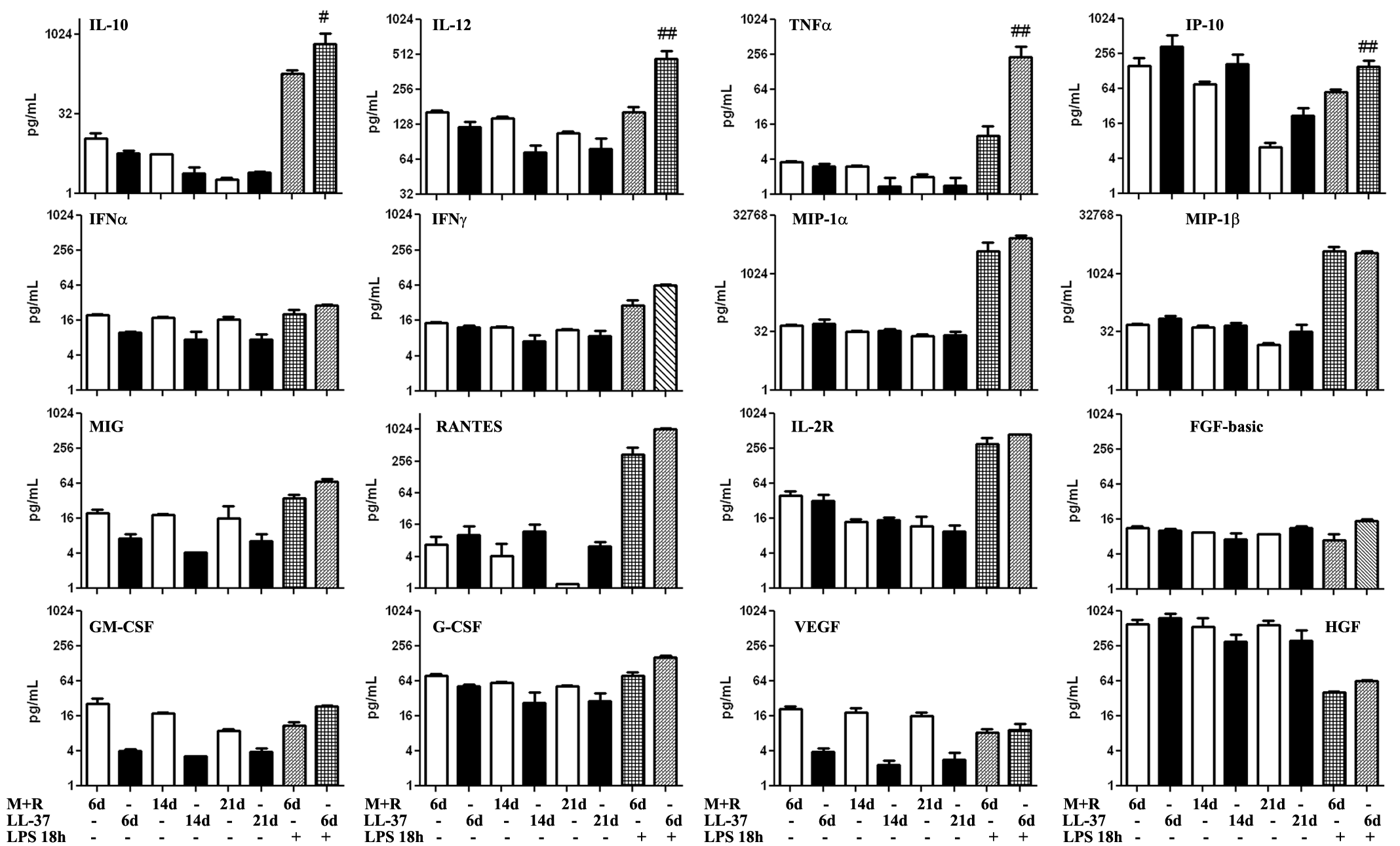

Supplement: Figure S2 — Comparison of cytokine release between LL-37-differentiated monocytes and M-CSF/RANKL-differentiated osteoclasts. Monocytes were incubated in the presence of 5 µM LL-37 or M-CSF/RANKL (both at 25 ng/mL). Cytokine levels in the supernatant of LL-37- and M-CSF/RANKL-differentiated monocytes were evaluated using the Human Cytokine 30-Plex antibody bead kit. In the experiment with LPS treatment, 6-day LL-37- or M-CSF/RANKL-differentiated monocytes were collected, resuspended at the concentration of 1×10^6^/mL, and incubated with 100 ng/mL LPS for 18 h. Cytokine levels were detected in the supernatant. Data (mean ± SE) were from three independent experiments performed. ## p<0.01 in comparison with M-CSF/RANKL (M+R)-differentiated monocytes treated with LPS for 18 h. (8.28 MB TIF) [file pone.0013985.s002.tif]

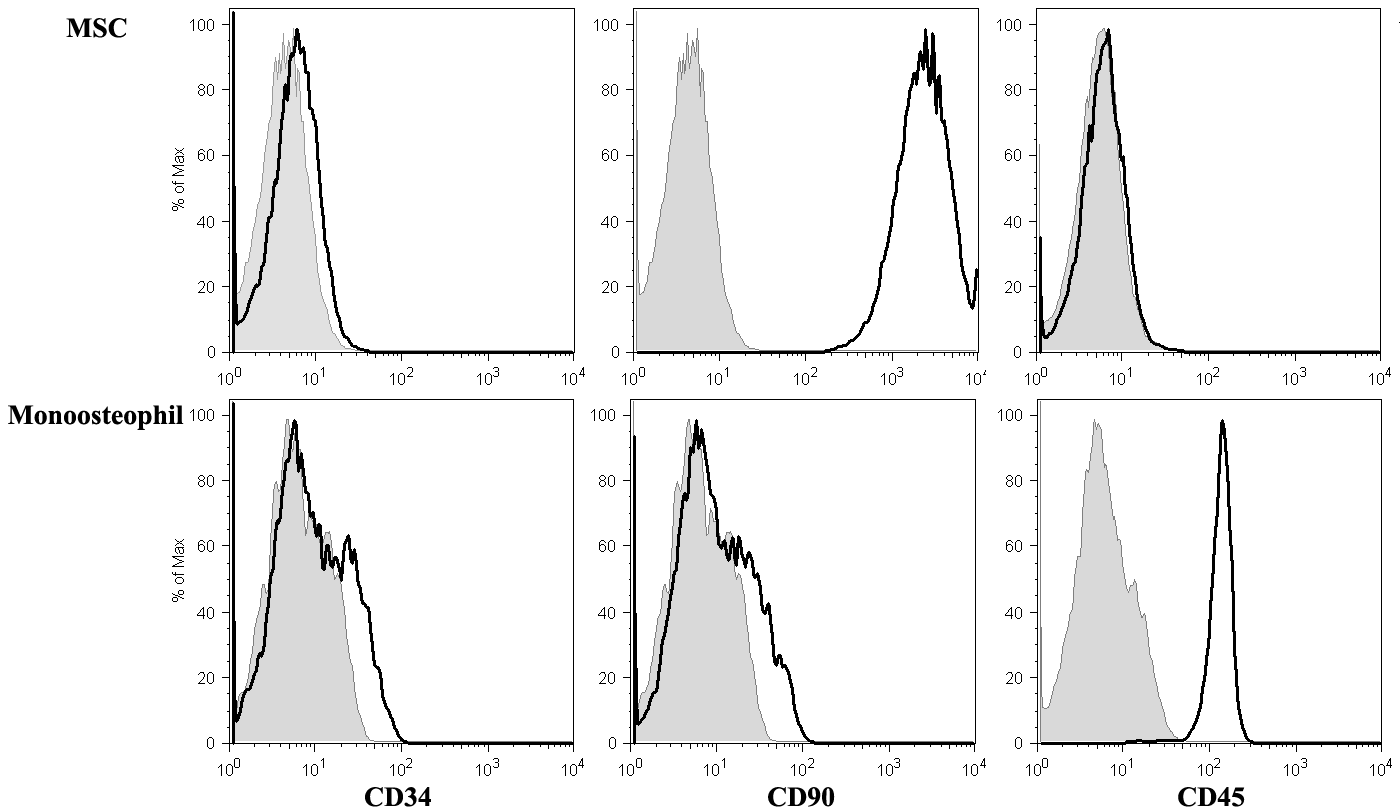

Supplement: Figure S3 — LL-37-differentiated monocytes show surface marker differences from mesenchymal stem cells (MSCs). MSCs and 6-day 5 µM LL-37-differentiated monocytes (monoosteophils) were harvested, stained with antibodies, and analyzed by using flow cytometry and Flowjo software. Data represent one of three independent experiments performed. (3.43 MB TIF) [file pone.0013985.s003.tif]

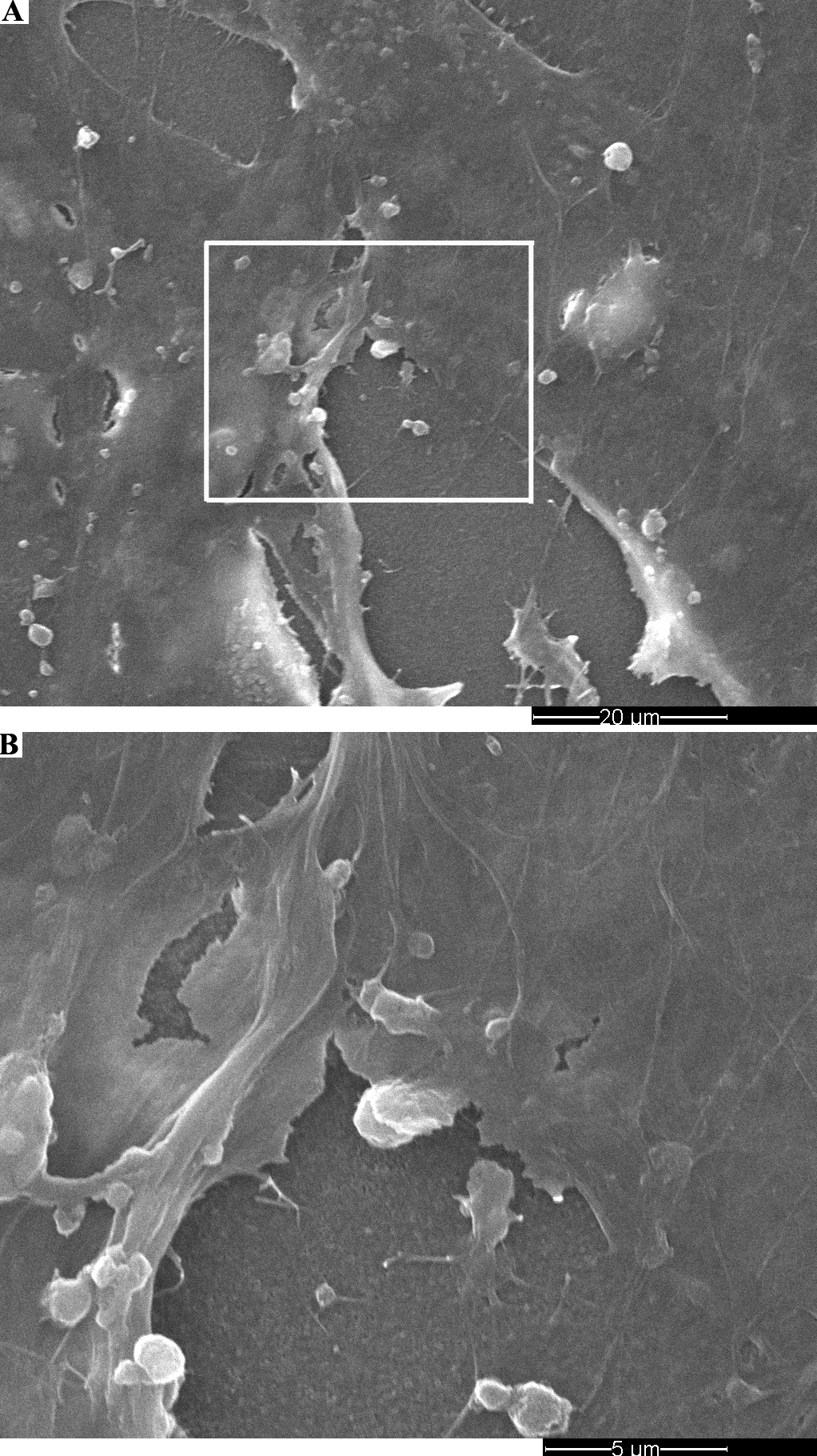

Supplement: Figure S4 — Differentiation of MSCs on osteologic disc. MSCs were incubated in the α-MEM supplemented with 10% FCS, 100 µM L-ascorbate-2-phosphate, 10−7 M dexamethasone on BioCoat™ Osteologic™ Discs in 5% CO2 atmosphere. After incubation for 4 weeks, built-up structures and cells were shown using SEM (A). The region shown in the rectangle was magnified to show further details (B). (1.89 MB TIF) [file pone.0013985.s004.tif]
