# Supplementary material for: Generation of Novel Bone Forming Cells (Monoosteophils) from the Cathelicidin-Derived Peptide LL-37 Treated Monocytes
Source: PLoS One. 2010 Nov 15;5(11):e13985. doi: 10.1371/journal.pone.0013985 (PMC2981577; doi:10.1371/journal.pone.0013985)
Supplement: Methods S1 — Supplementary methods. (0.02 MB DOC) [file pone.0013985.s005.doc]

**SUPPLEMENTARY INFORMATION**

**METHOD**

**von Kossa staining**

After 5 weeks of co-culture with control- or LL-37-differentiated monocytes, BioCoat™ Osteologic™ Discs were stained to demonstrated mineralization by using von Kossa staining according to BD Biosciences technical bulletin #444. Discs were photographed using phase contrast microscopy (magnification 200×).
