# Supplementary material for: Generation of Novel Bone Forming Cells (Monoosteophils) from the Cathelicidin-Derived Peptide LL-37 Treated Monocytes
Source: PLoS One. 2010 Nov 15;5(11):e13985. doi: 10.1371/journal.pone.0013985 (PMC2981577; doi:10.1371/journal.pone.0013985)
Supplement: Table S1 — Comparison of cytokine release of LL-37-differentiated cells for 6 days with medium, LPS, GM-CSF, and M-CSF differentiated macrophages (Mean±SEM). (0.05 MB DOC) [file pone.0013985.s006.doc]

**Supp Table 1. Comparison of cytokine release of LL-37-differentiated cells for 6 days with medium, LPS, GM-CSF, and M-CSF differentiated macrophages (Mean±SEM)**

|  | **Control** | **LPS** | **LL-37** | **GM-CSF** | **M-CSF** |
| --- | --- | --- | --- | --- | --- |
| **IL-4** | 9.0±0.68 | 15.22±0.13 | 7.16±0.59 | 11.82±0.98 | 11.07±0.95 |
| **IL-7** | 18.19±3.21 | 41.82±2.56 | 11.93±1.96 **#†‡** | 26.89±0.16 | 24.83±1.68 |
| **IFN-α** | 12.40±1.78 | 16.19±1.17 | 9.93±0.14 | 14.33±1.27 | 19.83±1.19 |
| **IFN-γ** | 13.47±0.69 | 15.94±0.49 | 12.13±0.88 | 13.5±0.47 | 14.67±1.06 |
| **GM-CSF** | 18.97±3.89 | 63.36±0.30 | 3.96±0.30***# ‡** |  | 25.2±5.51 |
| **MIP-1α** | 51.56±2.41 | 238.46±29.41 | 53.17±12.97 **#** | 72.20±6.18 | 41.62±2.63 |
| **MIP-1β** | 72.00±12.12 | 427.82±105.77 | 72.41±9.86 **#** | 70.42±19.71 | 47.26±1.46 |
| **MIG** | 18.14±1.34 | 27.58±0.93 | 7.16±1.31***#†‡** | 17.21±1.30 | 15.39±2.01 |
| **RANTES** | 7.46±2.79 | 10.88±0.95 | 10.24±3.87 | 5.42±1.85 | 10.19±1.38 |
| **MCP-1** | 15463.5±6862.6 | 9883.0±4145.9 | 7720.3±972.2 **‡** | 15362.1±7902.6 | 34236.5±1606.8 |
| **IL-2R** | 24.30±1.62 | 140.27±24.74 | 32.38±7.04**#** | 47.16±1.23 | 44.62±4.89 |
| **VEGF** | 8.67±0.87 | 61.16±12.70 | 3.88±0.41***#†‡** | 15.88±2.99 | 22.12±1.39 |
| **G-CSF** | 65.67±4.51 | 1217.43±81.09 | 51.79±3.38 **#** | 74.85±2.60 | 75.71±5.78 |
| **HGF** | 324.15±35.68 | 52.40±7.74 | 772.94±126.71**#** | 505.18±118.32 | 664.33±112.85 |
| **FGF-Basic** | 9.95±0.17 | 15.15±0.75 | 10.28±0.66 | 10.85±0.22 | 11.54±0.31 |

* p<0.05 in comparison with medium-macrophages; # p<0.05 vs LPS-macrophages; †p<0.05 vs GM-CSF-macrophages; ‡p<0.05 vs M-CSF-macrophages.
